# Antennal Sensitivity of Spotted Lanternflies, *Lycorma delicatula*: Differential Electrophysiological Responses of Males and Females to Compounds Derived from Host Plants and Conspecifics

**DOI:** 10.3390/insects15030162

**Published:** 2024-02-28

**Authors:** Hajar Faal, Miriam F. Cooperband

**Affiliations:** 1 Forest Pest Methods Laboratory, USDA–APHIS–PPQ, 1398 W. Truck Rd., Buzzards Bay, MA 02542, USA; 2 Tropical Research and Education Center, University of Florida, 18905 SW 280 St., Homestead, FL 33031, USA

**Keywords:** *Lycorma delicatula*, electroantennogram, semiochemicals

## Abstract

**Simple Summary:**

Electroantennography (EAG) was employed to screen the antennal sensitivity of male and female spotted lanternfly (SLF) fourth instars and adults to a range of 39 semiochemicals originating from their host plants and conspecifics, and semiochemicals were ranked in order of antennal response. An EAG study to a fixed dose of 50 ng indicated that the amplitude of antennal responses varied among stages and sexes. However, adult male antennae generated the largest EAG responses to identical chemicals. An EAG dose–response study of SLF antennae to a subset of compounds indicated that adult female antennae reach their saturation point at a higher dose than male antennae. Although EAG does not provide information on insect behavior, the current study reveals a spectrum of antennal sensitivity in response to different compounds and highlights the importance of chemical cues in SLF.

**Abstract:**

In herbivorous insects, antennae play a crucial role in chemical communication and orientation when locating host plants and mates. To evaluate antennal sensitivity in response to odor stimuli, electroantennography (EAG) has been a practical technique. In the current study of the invasive spotted lanternfly (SLF), *Lycorma delicatula* (Hemiptera: Fulgoridae), we evaluated and compared their antennal sensitivity to a series of volatile chemicals collected from their bodies, honeydew, and host plants. To do this, we exposed the antennae of SLF fourth-instar and adult males and females to individual chemicals at a fixed dose of 50 ng. Further, a series of dose–response tests were carried out within a range of 0.5 to 100 ng. Although the amplitude of antennal responses varied among stages and sexes, adult males generated the strongest antennal responses in both experiments. In dose–response experiments, increased doses of chemicals up to 50 ng revealed the saturation points except in adult females which required a higher dose (100 ng) to reveal the saturation point. Although EAG does not provide any information on behavioral responses, our results are consistent with the olfactory bioassays in previous publications in which adult males, not females, were attracted to natural volatiles of their conspecifics. EAG indicated a higher sensitivity of adult male antennae to odor stimuli, particularly conspecific volatiles, than female antennae and highlighted sexual differences in the perception of chemical cues in SLF.

## 1. Introduction

The invasive spotted lanternfly (SLF), *Lycorma* delicatula (Hemiptera: Fulgoridae), was first detected in Berks County, Pennsylvania, USA, in 2014 [1]. SLF are generalists that are known to feed upon at least 103 plant taxa, at least 56 of which are present in North America [2]. They threaten the grape industry, as well as impact agriculture and trade in numerous other industries in the US. SLF has been reported on fruit crops such as apple, cherry, and peach, as well as other hardwoods (black walnut, red maple, etc.) [3]. The value of the viticulture (grape), fruit tree, plant nursery, and timber industries is billions of dollars in Pennsylvania alone [4]. Therefore, as SLF spreads, it poses a serious threat to agricultural and forest industries across the country, while we still do not have a highly sensitive tool for survey and detection [5]. 

Semiochemicals are defined either as intraspecific signals (pheromones) or interspecific signals (kairomones and allomones) [6]. Insect perception of pheromones and kairomones plays a vital role in mediating their behavioral and physiological activities, such as host finding, feeding, social interactions, and mating [7]. Semiochemical-based lures in traps can provide precise species-specific and promising tools in integrated pest management [8]. Growers and IPM decision makers use semiochemical lures to trap and monitor the absence or presence of insect pests. They also are used to time pesticide applications when pest populations exceed threshold levels [9]. Additionally, semiochemical lures are essential tools used by government agencies to survey and monitor population spread or new pest introductions and guide large-scale management decisions. 

The electroantennogram (EAG) technique has been an essential tool in the identification of pheromones and plant volatiles mediating insect behaviors for many insect pests [10,11]. Insect antennae bear numerous olfactory chemoreceptors which can detect the presence of specific semiochemicals and transduce neural responses from the antennae to glomeruli in the antennal lobe of the brain [12]. EAG recordings sum the action potentials of the activated chemosensory neurons in the insect antennae that are sensitive to an odor stimulus [10,11]. The response is indicated by a sharp depolarization in action potential activity. The amplitude of the depolarization between the tip and the base of an insect’s antenna roughly corresponds to an insect’s antennal sensitivity, or tuning, to a particular compound. However, EAG activity only indicates what the antenna is capable of detecting, not how the brain processes this information, and, thus, does not predict a specific behavioral response [13]. Therefore, EAG-active compounds must be tested in behavioral studies to determine their functions.

The development of effective semiochemical-based monitoring tools for SLF is an objective of our research [14,15,16,17,18,19,20]. However, due to the large number of volatile compounds identified from SLF host plants, their honeydew, and body volatiles [16,18,19,20], there are too many individual compounds and potential blends to practically test, especially given the constraints of SLF being univoltine and lacking artificial rearing capabilities. EAG experiments revealed that a subset of these compounds could be detected by SLF antennae, with both male and female antennae capable of detecting the same set of compounds. A subset of those antennally active compounds was tested in bioassays and found to elicit behavioral responses, although they generated different responses among stages and sexes [16,18,19,20]. However, the list of antennally active compounds still contains an impractical number of compounds to test for attraction individually and in blends. Therefore, by investigating the relative antennal sensitivity to these compounds, we hope to gather information that could potentially narrow research efforts by prioritizing work on compounds to which the antennae are most sensitive. 

In the current study, we screened and compared the EAG responses of antennae from fourth-instar and adult SLF males and females to a series of authentic compounds that had been previously identified from host plants [18] and conspecific volatiles [16,20], presented at fixed doses. SLF males and females have similar diets and host plants [18,21], so we expected both male and female antennae to respond similarly to compounds from host plants. Based on previous dual-choice olfactometer studies, in which SLF were offered a choice between conspecific body volatiles or controls, where only males, but not females, were attracted to these volatiles [20], we hypothesized that SLF male antennae may be more sensitive, and, thus, produce larger EAG responses to conspecific compounds than female antennae. We also tested a subset of chemicals in an EAG dose–response experiment to look for differences in dose response among several compounds with different functional groups (alcohol, aldehyde, ester, and ketone). We found the activation thresholds of SLF antennae at the lowest and the highest doses of the selected chemicals, with saturation level defined as the highest dose at which the mean response was equal to or more than the next dose [22].

## 2. Materials and Methods

### 2.1. Insects 

**Figure 1:**
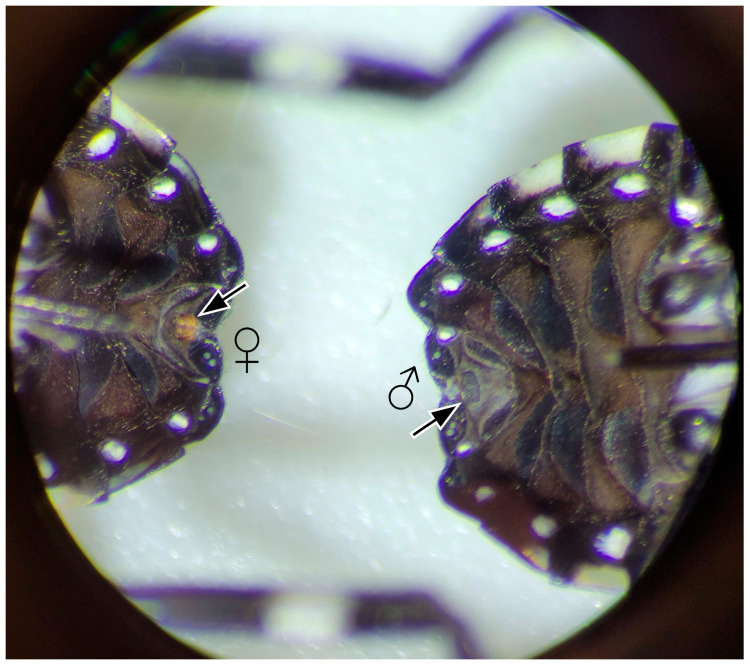
Ventral abdominal view of spotted lanternfly, *L. delicatula*, fourth-instar female (**left**) and male (**right**) under a dissecting microscope. Arrows point to the characteristic used to differentiate the sexes, showing the presence or absence of the developing light red female valvifers.

Fourth-instar and adult SLF were obtained from heavily infested field sites in Lehigh and Monroe Counties in Pennsylvania and Warren County in New Jersey between late-July and early-August 2021. Live insects were shipped to the insect containment facility located at the Forest Pest Methods Laboratory (FPML) in Buzzards Bay, Massachusetts, adhering to permit conditions (Pennsylvania Department of Agriculture (PDA) permit PP3-0123-2015 and U.S. Department of Agriculture (USDA) permits 526-23-107-88901 and P526P-20-03198). Inside containment, SLF fourth instars and adult males and females were reared in separate cages (47.5 × 47.5 × 93 cm, Bugdorm, Megaview Science Co., Ltd., Taichung City, Taiwan) containing potted tree-of-heaven (*Ailanthus altissima*) (40 cm tall, 4 L plant pots) plants, housing up to 20 or 30 SLF individuals each. Host plants were replaced as needed. All cages were kept at 23 ± 1 °C under natural daylight conditions in a containment greenhouse room, while supplemented with grow lights (TSL 2000 LED full spectrum, 2000 W, 25 × 100 cm coverage, Mars Hydro, Commerce, CA, USA) set for a 16:8 h light and dark photoperiod (light 06:00 to 20:00 h). Fourth-instar males and females were caged together, but they were sexed before conducting EAG experiments [23]. We used a dissecting microscope (Leica Microsystems, Model M80, Wetzlar, Germany) to distinguish fourth-instar females from males by observing light red valvifers at the distal end of their abdomen (Figure 1).

### 2.2. Chemicals

Authentic volatile compounds found in three odor sources were tested on the SLF antennae of fourth-instar and adult males and females. The sources were host plant volatiles [18], SLF honeydew volatiles [16], and SLF body volatiles from whole-body extracts [20]. The SLF honeydew-related volatiles were benzyl acetate (≥99%), 1-dodecanol (98%), 2-phenyl ethanol (≥99%), nonyl acetate (≥97%), decyl acetate (≥95%), and 2-ethylhexyl acetate (≥99%). The majority of chemicals tested were found in SLF body volatiles: octane (99%), undecane (99%), dodecane (99%), tridecane (99%), tetradecane (99%), 1-undecene (97%), 1-dodecene (95%), 1-tridecene (99%), (*Z*)-4-tetradecene (85%), 1-pentadecene (98%), hexanoic acid (≥99%), heptanoic acid (97%), octanoic acid (98%), hexanal (98%), heptanal (≥95%), octanal (99%), nonanal (≥95%), decanal (≥98%), 2-ethylhexanol (≥99%), 1-heptanol (98%), 1-octanol (≥99%), (*E*)-2-nonenal (≥95%), (*Z*)-6-nonenal (≥95%), (*E*)-2-decenal (≥97%), 2,3,-octanedione (75%). 6-Methyl-5-hepten-2-one (sulcatone) (≥98%) was a plant volatile. Methyl salicylate (≥99%) and 1-octen-3-ol (98%) were found in both host plant [18] and body [20] volatiles. Several ketones, 2-heptanone (98%); 2-octanone (98%); 2-nonanone (≥99%); an alcohol, 1-nonanol (98%); and an ester, isoamyl acetate (≥95%), were found in both SLF honeydew [16] and body [20] volatiles. Two chemicals, (*Z*)-4-tetradecene and 2,3,-octanedione, were synthesized by Tappey H. Jones (THJ) [20] and the rest of the authentic chemicals were purchased from Sigma-Aldrich (St. Louis, MO, USA), except (*E*)-2-nonenal (Fisher Scientific, Hampton, NH, USA).

### 2.3. Electroantennogram (EAG) Preparation and Recording

Hand-pulled, glass capillary saline electrodes were filled with Ringer’s solution (7.5 g/liter sodium chloride, 0.21 g/liter calcium chloride, 0.35 g/liter potassium chloride, and 0.2 g/liter sodium hydrogencarbonate) [18]. To prepare the SLF antenna for EAG recording, the insect head was carefully removed, and the ground electrode was inserted into the base of the head. The arista, with the tip removed, was inserted into the recording electrode. Antennal depolarization signals were amplified (Dam 50, World Precision Instruments, Sarasota, FL, USA) and then passed through an IDAC-2 acquisition controller (Syntech, Hilversum, The Netherlands) to a computer for data acquisition and analysis using GcEad/2014 software Version 1.2.5 (Syntech, Kirchzarten, Germany). 

EAG was conducted while coupled with an Agilent 7890B GC equipped with a flame ionization detector (FID), as previously described in Faal et al. [20]. In each test, 1 μL of solvent containing 100 ng of each of the compounds being tested was injected into the GC injector port in splitless mode (280 °C). The GC oven temperature was programmed from 60 °C for 2 min and subsequently increased to 250 °C at 10 °C/min. The program was stopped after the compounds eluted out of the column. The outlet of the GC column was split in a 1:1 ratio between the FID and the EAG; therefore, only half of the injected amount reached the SLF antenna. Each test injection was followed by a control injection of methyl salicylate (100 ng/µL) to ensure the antenna was still alive.

### 2.4. Antennal Sensitivity Recording

Two experiments were designed to determine the sensitivity of the SLF antennae to a series of selected chemicals from SLF honeydew and body volatiles and their host plant volatiles. 

#### 2.4.1. Fixed-Dose Relative Response

The antennal depolarization amplitudes in response to 39 authentic compounds at a fixed dose (50 ng) were quantified. For each compound, a total of twelve SLF antennae were used: three fourth-instar males, three fourth-instar females, three adult males, and three adult females. Each antenna was exposed to no more than two GC test injections. The size of the antennal depolarization in response to each chemical was directly measured as the maximum amplitude (mV) of change from the baseline using the Syntech Data Acquisition system [10]. The measurements are based on raw millivolt data, and we did not use any standards for normalizing EAG amplitudes. The average antennal response size and standard error (mV ± SE) are presented for each treatment. 

#### 2.4.2. Dose–Response

We aimed to find the saturation level of SLF antennae to six selected chemicals in four functional groups: 1-heptanol, sulcatone, benzyl acetate, 2-ethyl-hexanol, 2-nonanone, and nonanal. Compounds were prepared in two different blends designed to space the retention times of individual compounds, allowing the antenna to recover prior to exposure to each stimulus. In one blend, sulcatone, 2-nonanone, and benzyl acetate were presented, and 1-heptanol, 2-ethyl-hexanol, and nonanal were prepared in a different blend. These blends were prepared at four concentrations each an order of magnitude apart (0.1, 1, 10, and 100 ng/µL), and two additional concentrations (25 and 50 ng/µL) were added later due to the lack of antennal sensitivity at the two lowest doses of 0.1 and 1 ng/µL. A higher concentration of 200 ng/µL was added only for adult females to reach their antennal saturation point. Each blend also contained methyl salicylate as an internal standard at a constant concentration of 100 ng/µL. An antenna was tested by injecting 1 µL of the blend, at one of the concentrations listed above, into the GC, with half of each injection delivered to the insect antennae at the resulting doses: 0.05, 0.5, 5, 12.5, 25, 50, and 100 ng. The remaining half of each injection was simultaneously delivered to the FID. Antennal responses were recorded from male and female fourth instars (*n* = 4) and male and female adults (*n* = 5). Mean EAG amplitudes were plotted as a series of dose–response curves. The value of each response was standardized relative to the amplitude of response to methyl salicylate (100 ng/µL) present in the blend. Each insect antenna was tested no more than two times. 

### 2.5. Statistical Analysis

In the fixed-dose relative response experiment, data did not pass normality tests, and transformation did not improve data normalization. We used the Wilcoxon test (equivalent to Mann–Whitney) with a Bonferroni adjustment of α = 0.025 (JMP, version 15.2.0, SAS Institute, NC, USA) to compare the amplitudes of antennal responses between males and females of each stage, and between fourth instars and adults of each sex. In the dose–response experiment, log-transformed EAG amplitudes were analyzed using ANOVA and Tukey means separations to determine the saturation dose for a subset of six compounds, with α = 0.05 (JMP, version 15.2.0). Back-transformed data are presented.

## 3. Results

### 3.1. Antennal Sensitivity

#### 3.1.1. Fixed-Dose Relative Response

**Figure 2:**
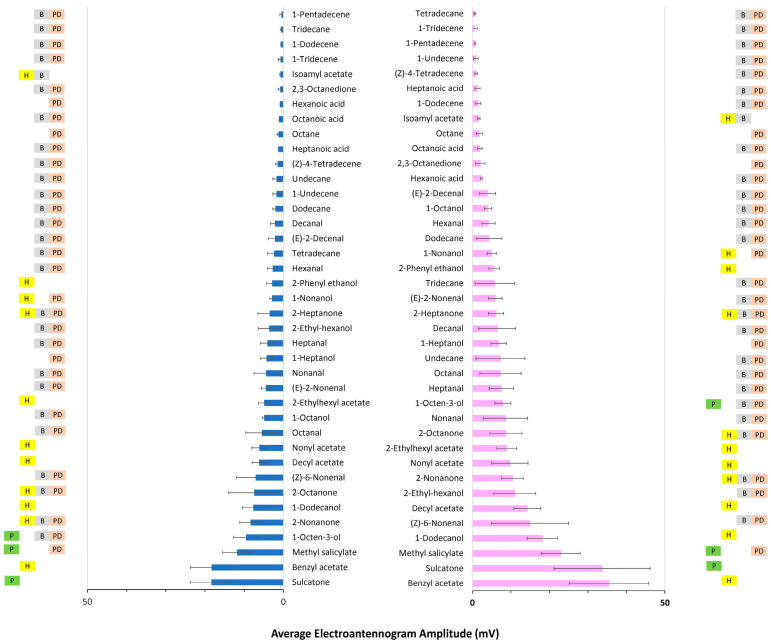
The average electroantennogram amplitudes (mV ± SE bars, *n* = 3) of fourth-instar spotted lanternfly, *L. delicatula*, male (left) and female (right) antennae to host plant (P), honeydew (H), body volatiles (B), or body volatiles found after photo-degradation (PD).

**Figure 3:**
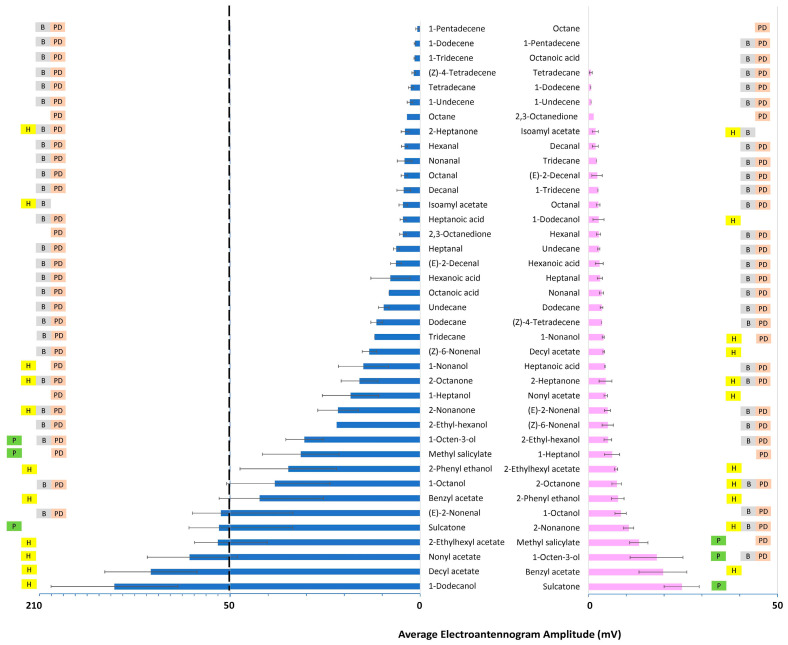
The average electroantennogram amplitudes (mV ± SE bars, *n* = 3) of adult spotted lanternfly, *L. delicatula*, male (left) and female (right) antennae to host plant (P), honeydew (H), body volatiles (B), or body volatiles found after photo-degradation (PD).

Almost all tested chemicals elicited measurable EAG responses in both sexes of SLF fourth instars (Figure 2) and adults (Figure 3). The exceptions were octane, octanoic acid, and 1-pentadecene, which did not elicit any measurable antennal activity in adult females (Figure 3). Although the antennae of both sexes could detect most of the chemicals, the intensity of antennal responses to some chemicals differed significantly between males and females. In fourth instars, on average, the female antennae generated larger EAG amplitudes than male antennae exposed to the same dose, with up to ten-fold differences (female average 11.0 ± 1.1 mV; male average 6.0 ± 0.6 mV; Wilcoxon Test: *df* = 1, *Z* = 4.62, *p* < 0.001). Interestingly, the sex with the higher antennal sensitivity to most compounds reversed in adults. The strongest EAG responses were elicited from adult male antennae (female average 7.9 ± 0.7 mV; male average 23.0 ± 2.6 mV; Wilcoxon Test: *df* = 1, *Z* = 5.37, *p* < 0.001). Interestingly, the average adult male antennal response to 1-dodecanol was 56 times larger than the average adult female response. The same chemicals elicited larger EAG amplitudes from adult male antennae than from female antennae, except (*Z*)-4-tetradecene and 1-tridecene, which generated two-fold stronger EAG responses from adult female than adult male antennae. The intensity of EAG amplitudes significantly increased from male fourth instars to adults (Wilcoxon Test: *df* = 1, *Z* = 7.96, *p* < 0.001), but not for females (Wilcoxon Test: *df* = 1, *Z* = 1.45, *p* = 0.145).

Both fourth-instar males and females produced the numerically strongest EAG responses to sulcatone (plant-derived), benzyl acetate (honeydew-derived), and methyl salicylate (plant- and body-derived) (Figure 2). In adults, the four strongest EAG responses in males were generated by honeydew-derived chemicals, 1-dodecanol, decyl acetate, nonyl acetate, and 2-ethylhexyl acetate. In adult females, sulcatone (plant-derived), benzyl acetate (honeydew-derived), 1-octen-3-ol (plant- and body-derived), and methyl salicylate (plant- and body-derived) elicited the largest EAG responses, respectively (Figure 3). 

#### 3.1.2. Dose–Response

**Figure 4:**
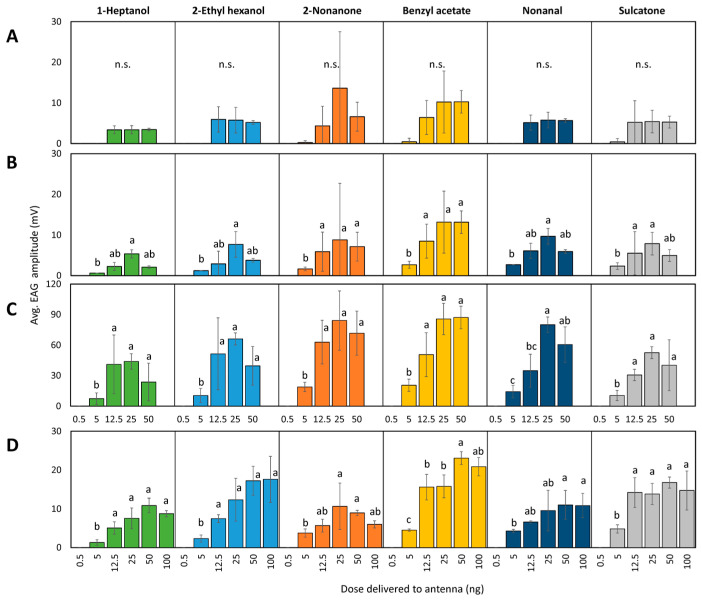
Antennal sensitivity of spotted lanternfly, *L. delicatula*, fourth-instar males (**A**), fourth-instar females (**B**), adult males (**C**), and adult females (**D**) to a subset of chemicals with different functional groups tested to determine a dose response. Tests consisted of 1 µL injections delivering either 0.5, 5, 12.5, 25, or 50 ng to the insect antennae in tests A, B, and C. For adult females (**D**), an additional dose delivered 100 ng to the antennae in order to reach its saturation point. The lowest dose all resulted in zero amplitudes and all zero amplitudes were excluded from the statistical analysis during log transformation; back-transformed data are shown. Bars in each section that do not share the same letter are significantly different (*p* < 0.05). Comparisons with no significant differences are marked with n.s. Note the difference in scale on the *Y*-axis for adult males (**C**).

Dose responses for a subset of compounds by each stage and sex are shown in Figure 4 and Table 1. No antennal responses were detected at the 0.5 ng dose for any sex or stage. EAG amplitudes in response to 5 to 50 or 100 ng chemical doses ranged from 0 to 13.6 mV in fourth-instar males, 0.6 to 13.2 in fourth-instar females, 7.4 to 88.2 mV in adult males, and 1.3 to 23.1 mV in adult females. No differences were found between doses for fourth-instar males (Figure 4A). On the other hand, fourth-instar females responded in a significant dose–response manner for all compounds, with the largest response to benzyl acetate and the lowest response to 1-heptanol. Fourth-instar females showed more sensitivity, with a significant EAG increase at 12.5 ng, to benzyl acetate, 2-nonanone, and sulcatone than to 1-heptanol, 2-ethyl hexanol, and nonanal. The saturation point for fourth-instar females to 1-heptanol, 2-ethyl hexanol, and nonanal was at 25 ng, whereas for 2-nonanone, benzyl acetate and sulcatone it was 12.5 ng (Figure 4B). 

Adult males showed a significant EAG increase at 12.5 ng to five of the six compounds tested, with 2-nonanone and benzyl acetate generating the largest responses and 1-heptanol generating the smallest responses, and saturation points for all six compounds at 25 ng (Figure 4C). Adult females produced significant dose responses to all six compounds, with sensitivity increasing significantly at 12.5 ng to 1-heptanol, 2-ethyl hexanol, benzyl acetate, and sulcatone, at 25 ng for 2-nonanone, and nonanal at 50 ng (Figure 4D). Of the subset of six compounds, the largest and smallest EAG responses in adult females were generated by benzyl acetate and 1-heptanol, respectively (Figure 4D). Saturation points in adult females appeared to occur at 50 ng for 1-heptanol, 2-ethyl hexanol, benzyl acetate, and nonanal, at 25 ng for 2-nonanone, and at 12.5 ng for sulcatone. 

## 4. Discussion

The antennal sensitivities of male and female fourth-instar and adult SLF were compared for 39 volatile compounds related to SLF host plants, honeydew, and bodies using GC-EAG. Wang et al. [24] found that SLF antennae have up to six types of chemosensory structures, dominated by sensilla placodea or plate organs on their antennae, and that the number and size of these sensilla increase with increasing nymphal stage. They also found sexual dimorphism in SLF antennal sensilla, in that adult females had more and larger sensilla placodea than adult males. Our results show that the strongest antennal responses were generated by adult males, followed by adult females, fourth-instar females, and fourth-instar males. The EAG amplitudes of adult males were 20 times greater than fourth-instar males, which was expected because the number and size of antennal sensilla on adult antennae was greater than on nymphs [24]. However, a similar pattern was not observed in antennal responses of females, which was surprising. Considering that adult SLF females have a larger number of antennal sensilla than adult males [24], it was also unexpected that a fixed dose of identical chemicals elicited much larger EAG responses in adult males than in females.

Differences in antennal sensitivity between males and females have been observed in other insects as well [25,26,27]. Differences may be due to differing requirements in chemical and behavioral ecology between the sexes [28], for example, males locating females for mating and females locating oviposition sites. Several other hypotheses have been suggested as possible mechanisms for sexual differences in antennal responses, including differences in the electrical resistance of the antennae, the number and density of olfactory sensory neurons and their sensitivity, or even the specificities of receptor cells in sensilla [10,29,30,31]. 

Chemosensory sensilla on male and female antennae have been described in detail for SLF [24]. Their antennae have a short ring-like scape at the base, a large, round pedicel which houses numerous chemosensory plate organs, and a narrow flagellum which is composed of a basal bulb and a threadlike apical arista [24]. The basal bulb carries chemoreceptive sensory plate organs only during the first, second, and third instars. The majority of the chemosensory plate organs appear on the pedicel starting in the second instar. The number and size of these sensilla increase with each SLF nymphal instar and are maximized in adults [24]. A significant sexual dimorphism occurs in the sensilla types and numbers, sensillum basal diameter, and number of sensory plate organs on adult SLF antennae [24]. The antennal pedicel of adult females has significantly more sensilla and larger sensilla than their male counterparts [24]. In the current study, adult males produced stronger EAG responses to plant- and conspecific-derived chemicals than females, which was surprising considering that it is the female that features more chemosensory antennal sensilla. Therefore, we cannot attribute our EAG results to the difference in the general number of peripheral sensory structures located on adult male and female SLF antennae [24]. Each sensillum contains one or multiple olfactory sensory neurons which have odorant binding protein receptors on their surface that bind to specific odor molecules and result in the depolarization of the neuron [32]. Single sensillum recordings can provide information about the specific compound or compounds that an individual sensillum on the antenna is capable of detecting [33]. Although sensilla may be morphologically similar, each sensillum on the antenna has olfactory receptors tuned to detect specific compounds. Differences in the numbers of sensilla that are receptive to specific compounds could be responsible for differences in the antennal response signal strength, a topic which single sensillum recordings on SLF antennae could potentially elucidate.

Most of our knowledge on deciphering the neuronal circuits that mediate insect behavioral activities has focused on the electrophysiological activity of antennal neurons to authentic standards derived from their host plant and conspecific volatiles. We do not know why male SLF generated very large EAG responses relative to females, whose chemoreceptive sensilla are larger and more numerous [24]. It is likely due to specific sex-related qualitative differences in olfactory physiology or additional modifications at all levels of the neural pathway controlling sensitivity to odors. Wang et al. [24] described the morphological characters of SLF antennae and the possible function for each sensillum type and demonstrated the sexual dimorphism in the number and size of sensilla in adult males and females. In the current study, one arista was used for EAG recording while it was still attached to the whole insect head. Thus, it is possible that olfactory sensilla on the head were also involved in the recording. Hao et al. [34] described the distribution of sensilla located on the proboscis of SLF females. They did not describe their male counterparts due to lack of obvious structural or fine-structural differences between the male and female proboscis. Currently, a more detailed understanding of SLF chemical detection at the molecular and cellular levels remains unknown. 

Faal et al. [15,20] showed the attraction of SLF male adults, not female adults, to their conspecific body volatiles. The differences in attraction between adult males and females may be explained by differences in their antennal sensitivity to identical compounds [29]. In the dose–response experiment, the maximum EAG responses in adult female SLF usually occurred at a higher dose of 50 ng compared to 25 ng for adult males. The exception for adult females was 2-nonanone, which also had a maximum response at 25 ng. However, antennal responses to chemicals cannot inform us about their behavioral function. For that, behavioral evidence, such as in olfactory bioassays or field studies, must be collected. EAG has been used for qualitative measurements, as it provides an overall view of the change in the electrical potential from insect antennae in response to the chemical stimulus [10,35], but qualitative measurements of amplitude may depend on the length of insect antennae inserted into the recording electrode [36], the connection strength, and insect vitality [10,11]. Therefore, in the absence of other data, a small EAG response cannot necessarily be interpreted as a poor ability of the insect to detect or respond to a chemical. Relative amplitudes of multiple compounds may provide additional context in combination with behavioral data. The order of EAG intensity to compounds can provide clues, but cannot alone convey their attractiveness or other behavioral functions [37]. 

SLF are known to detect a wide range of plant-, honeydew-, and body-derived volatiles, the natural blends of which result in attraction [15,16,18,19,20]. With the goal of developing attractive lures for detection, survey, and mitigation, it is necessary to distinguish which combination of those compounds produces the most potent blend to elicit the desired behavior. Laboratory and field testing for attraction to blends of plant volatiles has been conducted [18,19], and from 2015 to 2023, targeting various SLF stages, we conducted over 50 field tests of blends of both plant- and SLF-derived attractants without finding a clear improvement to methyl salicylate alone [Cooperband, unpublished]. Without the mass rearing capabilities for SLF, field and laboratory testing specifically for *sexual* attractants is constrained to roughly a 3-week window per year using wild-caught insects. With over 40 antennally active compounds described for SLF, the possible number of blends and ratios are far too many to test for attraction in that timeframe. Using GC-EAG, we can potentially focus our attention on the compounds that are more promising. This study sought to describe the differences in SLF antennal responses to an array of semiochemicals, and found that the amplitude of EAG responses differed depending on the compound, stage, and sex. These results confirm that SLF antennae are equipped with olfactory receptors capable of sensing a range of semiochemicals, and reveal that unknown differences between male and female antennae result in different strengths of antennal signals reaching the brain. We have demonstrated repeatedly, using different collection techniques, that male SLF distinguish between volatiles collected from male vs. female SLF [15,16,20], but the compounds responsible for these behaviors have not been identified yet. Differences in EAG activity between adult males and females revealed a greater sensitivity to honeydew-derived volatiles by male antennae and to plant-derived compounds by female antennae. Differences in antennal sensitivity might align with their behavior and ecology. SLF adult males oriented to conspecific body and honeydew volatiles, whereas adult females showed a trend of attraction to the volatiles from female honeydew, but not to their conspecific body volatiles [15,16,20]. SLF adults, significantly, were attracted to three compounds, (*Z*)-3-hexenol, (*E*,*E*)-α-farnesene, and methyl salicylate, commonly found in volatiles from their preferred host, *A. altissima* [18,19]. SLF may start forming aggregations using plant volatiles as cues, whereas males may use cues from honeydew and sex-specific volatiles in mate-location. For this, each may need a higher olfactory sensitivity tuned to these specific tasks. Thus, the current study highlights the important functions of SLF chemical cues in locating host plants and conspecifics for feeding, aggregation, and mating. 

## 5. Conclusions

The antennae of male and female SLF detect a wide range of semiochemicals originating from their host plants and conspecifics. Male and female antennae from fourth instars and adults were tested, and adult male antennae generated the largest EAG responses to identical chemicals at a fixed exposure dose. Adult males produced the highest EAG amplitudes in response to honeydew-derived chemicals and adult females produced the highest EAG amplitudes in response to plant-derived chemicals. A dose–response study of SLF antennae to a blend of compounds indicated that adult female antennae reach their saturation point at a higher dose than male antennae. This is consistent with previous bioassay results in which identical amounts of natural body volatiles were presented to both sexes, and while males were significantly attracted, female attraction was approaching significance and might have been significant if presented at a higher dose. How SLF males have a higher antennal sensitivity to lower doses of honeydew volatiles compared to females whose antennae numerically have more olfactory sensilla is a topic that would require single sensillum recordings to better understand the sensitivity and selectivity of individual sensilla on insect antennae [38].

## Figures and Tables

**Table 1:** ANOVA results comparing the log-transformed antennal responses of spotted lanternfly, *L. delicatula*, fourth-instar males and females, and adult males and females to a subset of six chemicals, comparing the doses within each compound, as well as a comparison of all six compounds to each other. The degrees of freedom (d.f.) are shown for the model and error, respectively, as well as the number of EAG recordings (N) used in each statistical test. *p*-Values less than 0.05 indicate that the antennal responses to different doses were significantly different.

Stage	Sex	Compound	*p*-Value	F-Ratio	d.f.	N
Fourth instar	Male	1-Heptanol	0.956	0.05	2, 9	12
		2-Ethyl-hexanol	0.980	0.02	2, 9	12
		2-Nonanone	0.255	1.61	3, 9	13
		Benzyl acetate	0.081	3.11	3, 9	13
		Nonanal	0.786	0.25	2, 9	12
		Sulcatone	0.403	1.09	3, 9	13
	Female	1-Heptanol	0.013	5.80	3, 11	15
		2-Ethyl-hexanol	0.012	5.93	3, 11	15
		2-Nonanone	<0.001	14.72	3, 12	16
		Benzyl acetate	<0.001	28.23	3, 12	16
		Nonanal	0.039	3.85	3, 12	16
		Sulcatone	0.002	8.96	3, 12	16
Adult	Male	1-Heptanol	0.002	8.31	3, 13	17
		2-Ethyl-hexanol	0.001	12.34	3, 12	16
		2-Nonanone	<0.001	21.75	3, 13	17
		Benzyl acetate	<0.001	19.19	3, 13	17
		Nonanal	<0.001	15.04	3, 13	17
		Sulcatone	<0.001	12.90	3, 13	17
	Female	1-Heptanol	<0.001	20.42	4, 10	15
		2-Ethyl-hexanol	<0.001	17.87	4, 10	15
		2-Nonanone	0.026	4.43	4, 10	15
		Benzyl acetate	<0.001	67.05	4, 10	15
		Nonanal	0.034	4.03	4, 10	15
		Sulcatone	0.001	13.43	4, 10	15

## Data Availability

The data presented in this study are openly available in Figure 1, Figure 2 and Figure 3.
